# Sodium fluoride induces apoptosis in cultured splenic lymphocytes from mice

**DOI:** 10.18632/oncotarget.12081

**Published:** 2016-09-16

**Authors:** Huidan Deng, Ping Kuang, Hengmin Cui, Lian Chen, Jing Fang, Zhicai Zuo, Junliang Deng, Xun Wang, Ling Zhao

**Affiliations:** ^1^ College of Veterinary Medicine, Sichuan Agricultural University, Ya'an, China; ^2^ Key Laboratory of Animal Diseases and Environmental Hazards of Sichuan Province, Ya'an, China

**Keywords:** NaF, splenic lymphocytes, apoptosis, Bcl-2 family, caspases, Immunology and Microbiology Section, Immune response, Immunity

## Abstract

Though fluorine has been shown to induce apoptosis in immune organs *in vivo*, there has no report on fluoride-induced apoptosis in the cultured lymphocytes. Therefore, this study was conducted with objective of investigating apoptosis induced by sodium fluoride (NaF) and the mechanism behind that in the cultured splenic lymphocytes by flow cytometry, western blot and Hoechst 33258 staining. The splenic lymphocytes were isolated from 3 weeks old male ICR mice and exposed to NaF (0, 100, 200, and 400 μmol/L) *in vitro* for 24 and 48 h. When compared to control group, flow cytometry assay and Hoechst 33258 staining showed that NaF induced lymphocytes apoptosis, which was promoted by decrease of mitochondria transmembrane potential, up-regulation of Bax, Bak, Fas, FasL, caspase 9, caspase 8, caspase 7, caspase 6 and caspase 3 protein expression (*P* < 0.05 or *P* <0.01), and down-regulation of Bcl-2 and Bcl-xL protein expression (*P* <0.05 or *P* <0.01). The above-mentioned data suggested that NaF-induced apoptosis in splenic lymphocytes could be mediated by mitochondrial and death receptor pathways.

## INTRODUCTION

Fluorine is one of the essential trace elements for human health [1]. However, excessive fluoride intake can cause serious tissue damage and lead to multiple organ dis-function [2, 3], which depends not only on the concentration and exposed duration [4, 5], but also on the absorption capacity, age, and nutritional status of the individual [6]. It has been demonstrated that fluoride can induce skeletal fluorosis and soft tissue damage [7-9]. Also, our previous studies have proved that fluorine can induce cytotoxicity, immunotoxicity, oxidative damage and pathological injury in the thymus, spleen, bursa of Fabricius, cecal tonsil, liver, kidney, peripheral blood and intestine of broiler chickens [10-36].

In recent years, fluoride has been shown to induce immunotoxicity *in vivo* and *in vitro*. Animal studies have showed that fluoride reduces T cell and B cell numbers [27, 37, 38], and increases G0/G1 thymocytes and splenocytes population in young broiler chickens [27, 28]. However, analysis of fluoride effects on cell cycle phases in cultured rat osteoblasts shows an increase number of cells at S phase and a decrease in cells at G2/M phase, while the cells in Go/G1 are not changed [39]. Refsnes et al. [40] have reported that fluoride can induce IL-6 and IL-8 production in human epithelial lung cells, while the concentration of IL-4, IL-6, TNF-α, and IFN-γ in the cecal tonsil and intestinal mucosa of broilers have been found to be decreased in the high dietary fluorine groups *in vivo* studies [29, 31]. Luo et al. and Liu et al. [13, 16] have reported that IgM and IgG contents in the cecal tonsil and intestines are decreased in high fluorine groups, which demonstrates that high dietary fluorine can impact the humoral immunity. In cecal tonsil of young chickens [30], the percentage of CD3+, CD3+ CD8+ and CD3+ CD4+ T lymphocytes are also decreased in high fluorine groups. Meanwhile, study *in vitro* has also reported [37] that fluorine ion can suppress T lymphocytes proliferation, IL-2 and IL-6 production, and decrease the CD4+ lymphocytes proportions.

Apoptosis, or programmed cell death, is an essential physiological process that plays a critical role in regulating cell growth and immune response *via* gene expression and/or protein activity [41, 42]. It has been reported that fluoride can induce cell apoptosis [43]. Our previous studies have found that fluorine can induce apoptosis by altering Bcl-2 and Bax expression in the splenic lymphocytes of broiler chickens [19, 25]. However, the underlying mechanisms of the fluoride-induced apoptosis of lymphocytes are still unknown.

Therefore, in order to reveal possible pathway involved in sodium fluoride (NaF)-induced apoptosis in the immune system, this *in vitro* study was conducted to investigate the apoptosis and apoptosis-related proteins in the cultured splenic lymphocytes from mice. The results would provide new experimental evidences for understanding the effect mechanism of NaF on splenic immune function.

## RESULTS

### NaF reduced viability of the splenic lymphocytes

**Figure 1:**
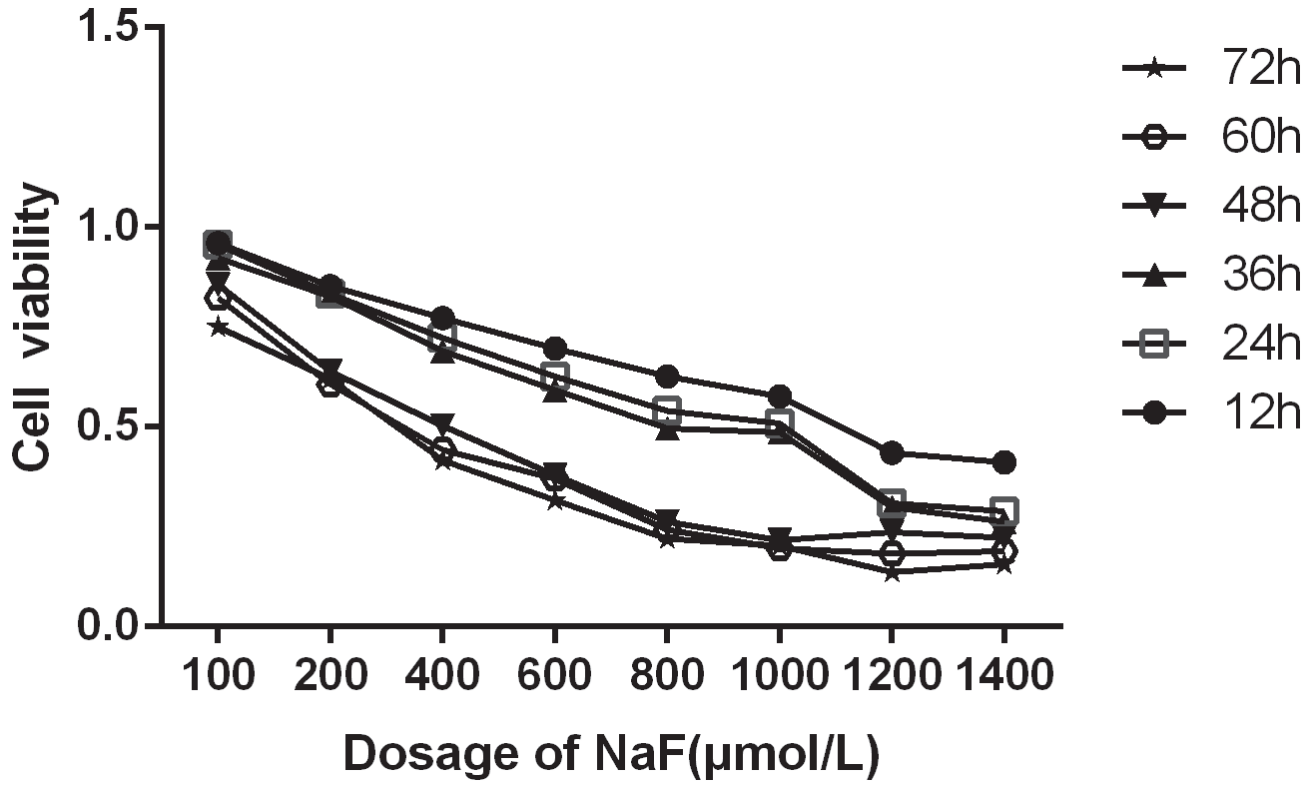
Cell viabilities of cultured splenic lymphocytes The cell viability was determined using a CCK8 assay as described in the methodology section. The splenic lymphocytes were incubated with different NaF concentrations at different times. Data were analyzed by the variance (ANOVA) test of the SPSS 19.0 software.

CCK-8 assay was performed to test the cytotoxic effect of NaF on splenic lymphocyte viability. As shown in Figure 1, the viability of splenic lymphocytes was significantly decreased (*P* < 0.05) at 100-800 μmol/L NaF exposure for 12 h-72 h. And the IC50 value of NaF-decreased cell ability was estimated to be 400 μmol/L NaF for 48 h. Approximately 70% of cells were survived at 400 μmol/L NaF exposure for 24 h. Based on these findings, treatment of 100 (low-dose group, LG), 200 (medial -dose group, MG) and 400 (high-dose group, HG) μmol/L NaF for 24 h and 48 h was selected for the following studies.

### Dispensable of ROS activity in NaF induced cell apoptosis

**Figure 2:**
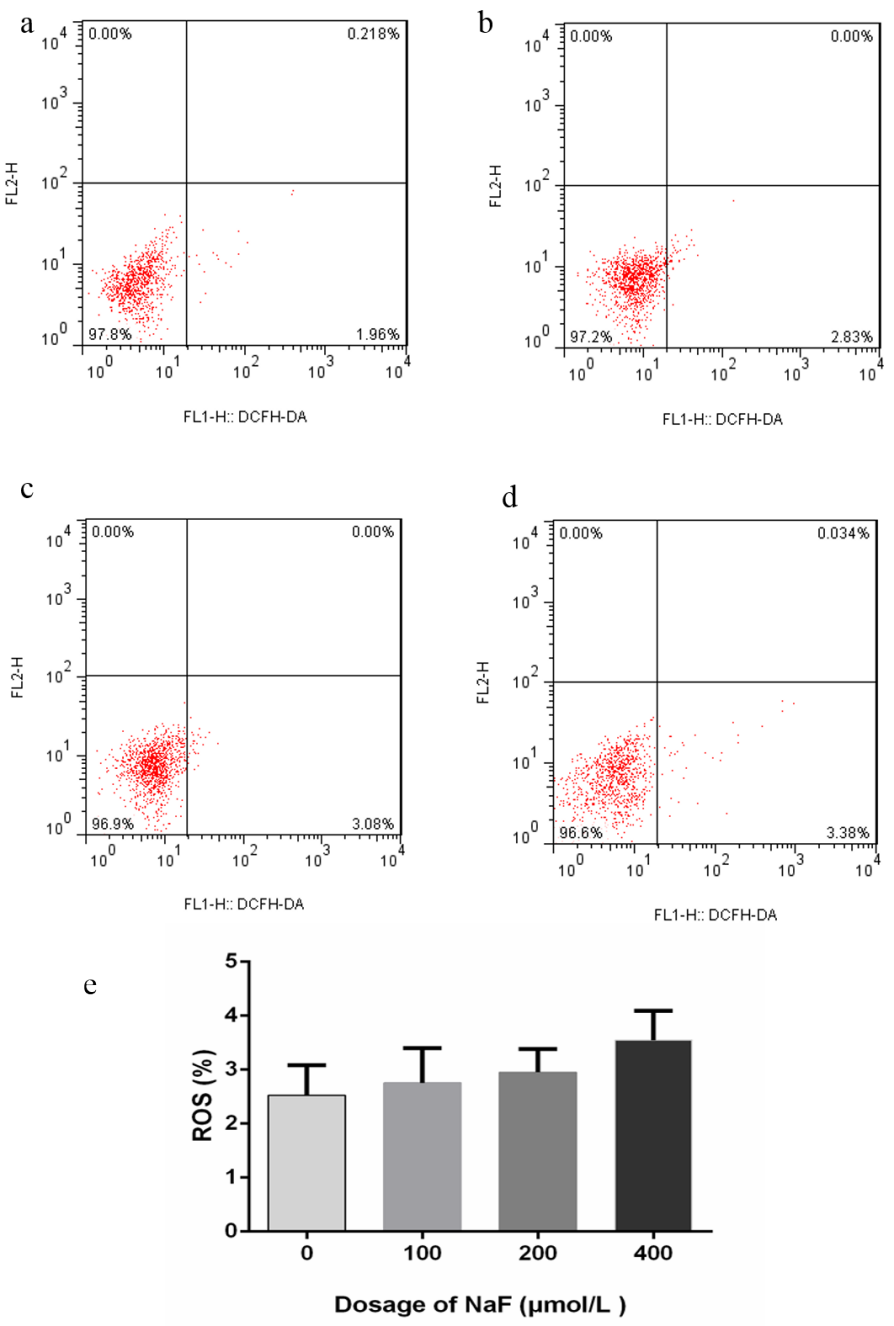
Effect of NaF on ROS generation at 24 h **a.-d.** Two-dimension scatter plots depicting distribution of cells positively stained for DCFH-DA. (a) CG, (b) LG, (c) MG and (d) HG. e. Quantitative analysis of ROS generation. Data are presented with the means ± standard deviation, * *p* < 0.05, ** *p* < 0.01, compared with the control group. Data were analyzed by the variance (ANOVA) test of the SPSS 19.0 software.

**Figure 3:**
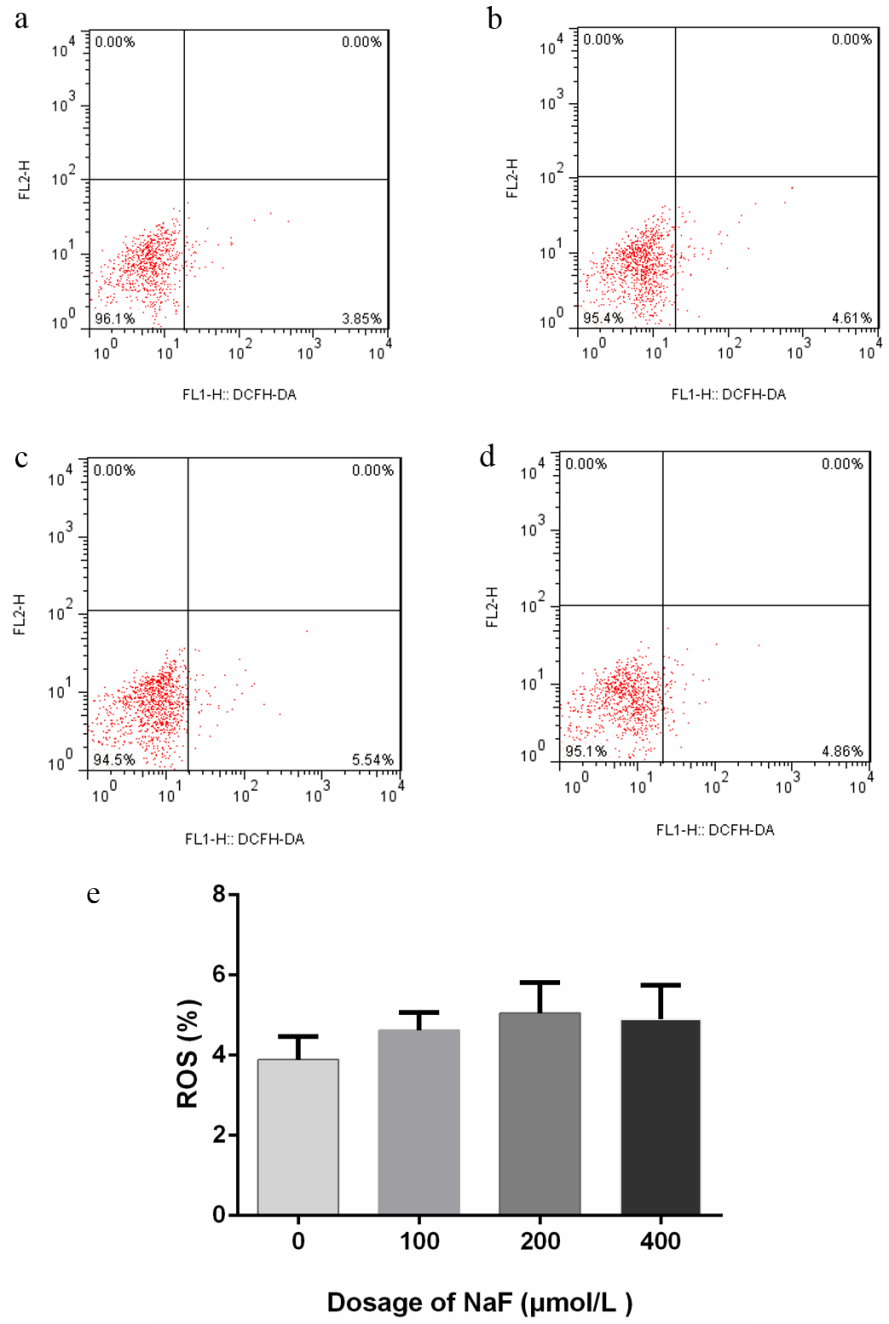
Effect of NaF on ROS generation at 48 h **a-d.** Two-dimension scatter plots depicting distribution of cells positively stained for DCFH-DA. (a) CG, (b) LG, (c) MG and (d) HG. e. Quantitative analysis of ROS generation. Data are presented with the means ± standard deviation, * *p* < 0.05, ** *p* < 0.01, compared with the control group. Data were analyzed by the variance (ANOVA) test of the SPSS 19.0 software.

To determine whether ROS is involved in NaF-induced cell death of splenic lymphocytes, the level of ROS production was measured by using DCFH-DA. Figure 2 and 3 showed that the concentration of NaF did not affect the ROS level in splenic lymphocytes. Overall, NaF induced apoptotic cell death in splenic lymphocytes was not through ROS pathway.

### Effects of NaF on mitochondria transmembrane potential (MMP) in splenic lymphocytes

**Figure 4:**
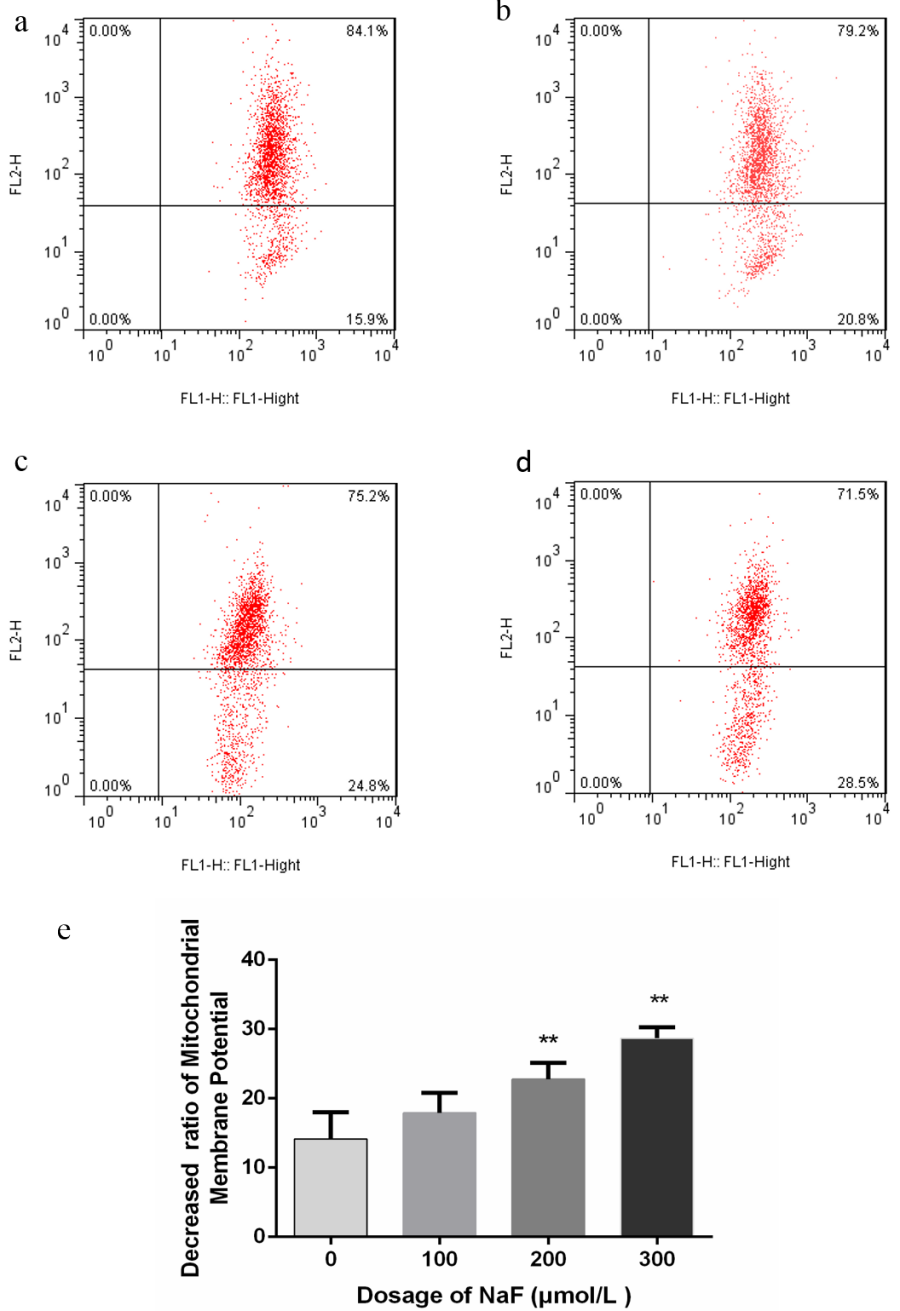
Effect of NaF on the MMP Δ*ψm* of splenic lymphocytes at 24 h **a.-d.** Assessment of mitochondrial membrane potential of lymphocytes with JC-1 staining by flow cytometry method. (a) CG, (b) LG, (c) MG and (d) HG. e. Quantitative analysis of the percentage of lymphocytes with green fluorescence. Data are presented with the means ± standard deviation, * *p* < 0.05, ** *p* < 0.01, compared with the control group. Data were analyzed by the variance (ANOVA) test of the SPSS 19.0 software.

**Figure 5:**
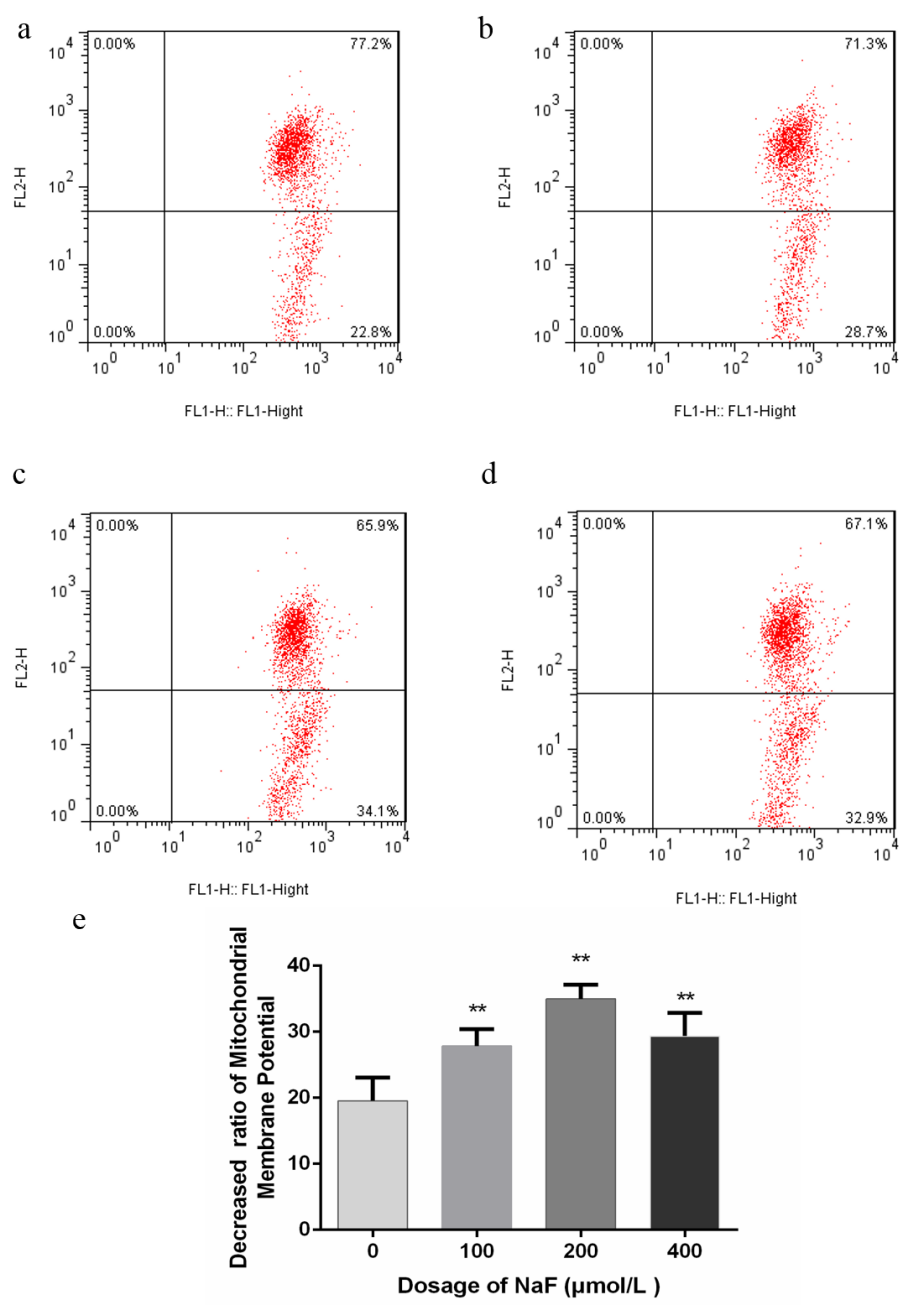
Effect of NaF on the MMP Δ*ψm* of splenic lymphocytes at 48 h **a.-d.** Assessment of mitochondrial membrane potential of lymphocytes with JC-1 staining by flow cytometry method. (a) CG, (b) LG, (c) MG and (d) HG. e. Quantitative analysis of the percentage of lymphocytes with green fluorescence. Data are presented with the means ± standard deviation, * *p* < 0.05, ** *p* < 0.01, compared with the control group. Data were analyzed by the variance (ANOVA) test of the SPSS 19.0 software.

It was well established that the induction of apoptosis was associated with the perturbation of mitochondrial functions. Here, we examined the changes of MMP by using the fluorescent dyes JC-1. Cells in right upper quadrant correspond to red fluorescence signal intensity, and cells in right lower quadrant correspond to green fluorescence signal intensity. As shown in Figure 4 and 5, the percentages of lymphocytes depolarized with collapse of the Δψm were significantly increased (*p* < 0.01) in the MG and HG in the presence of NaF for 24 h. Moreover, NaF treatment for 48 h decreased (*p* < 0.01) the percentage of lymphocytes depolarized with collapse of the Δψm in the LG, MG and HG when compared to CG. Those results suggested that NaF caused the MMP change, which could lead to the apoptosis of splenic lymhoocytes.

### NaF caused apoptosis in splenic lymphocytes

**Figure 6:**
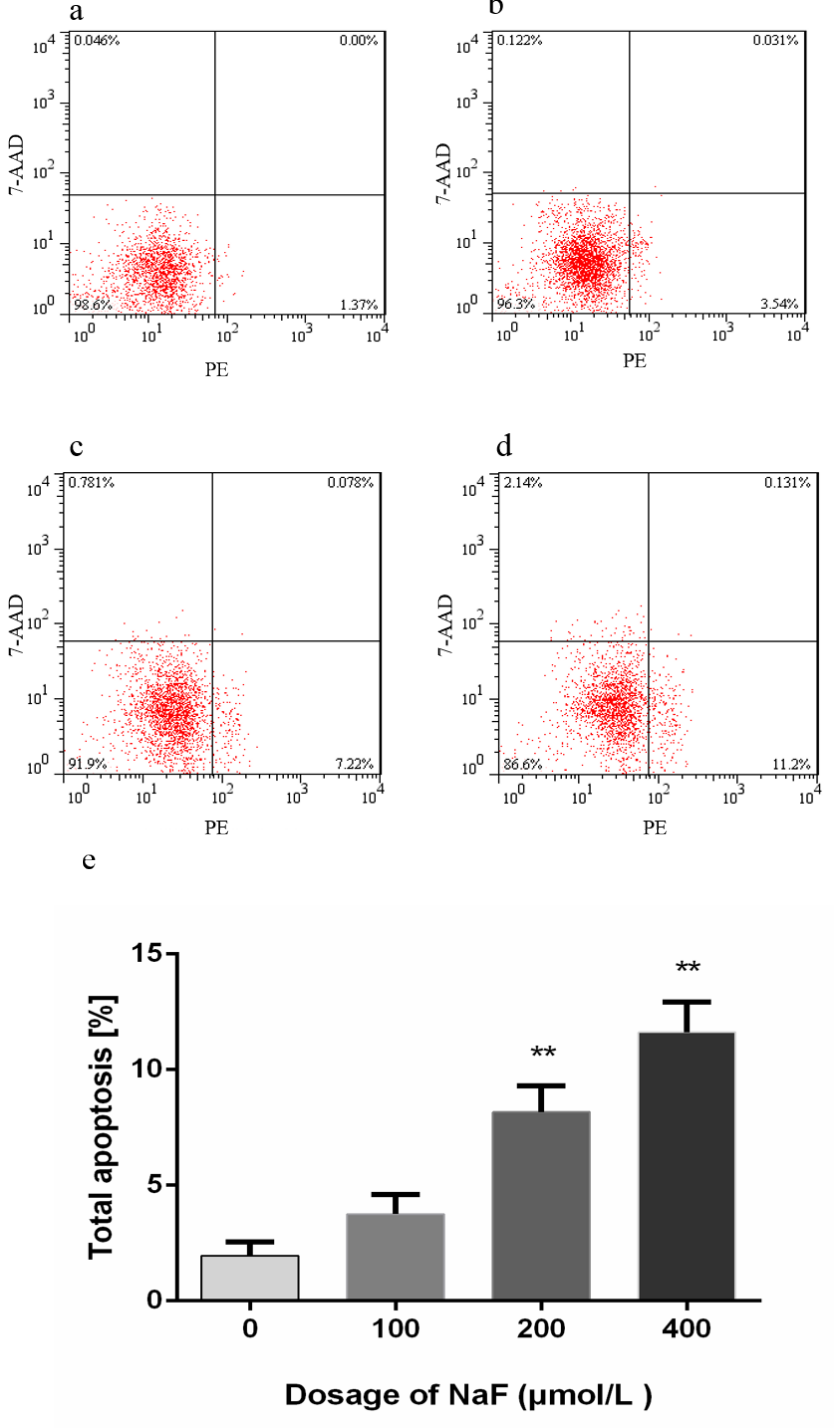
Effect of NaF treatment on apoptosis of cultured splenic lymphocytes at 24 h **a.-d.** Two-dimension scatter plots depicting distribution of cells positively stained for Annexin V-PE/7-AAD. (a) CG, (b) LG, (c) MG and (d) HG. Cells in lower left quadrant of each picture correspond to normal cells. Cells in right lower quadrant correspond to early apoptotic cells. Cells in right upper quadrant correspond to late apoptotic. Cells in left upper quadrant correspond to dead cells. **e.** Quantitative analysis of total apoptotic lymphocytes. Data are presented with the means ± standard deviation, * *p* < 0.05, ** *p* < 0.01, compared with the control group. Data were analyzed by the variance (ANOVA) test of the SPSS 19.0 software.

**Figure 7:**
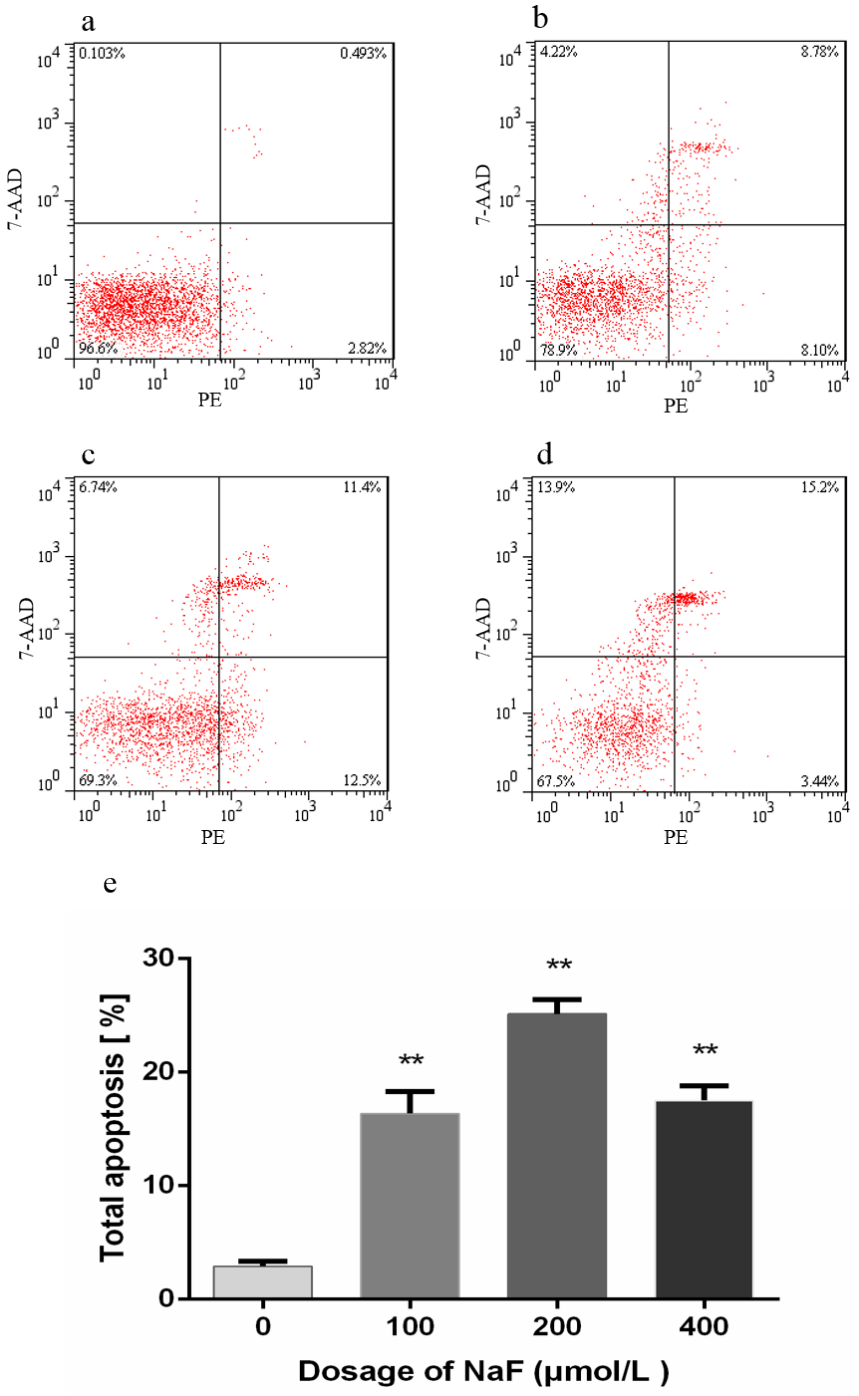
Effect of NaF treatment on apoptosis of cultured splenic lymphocytes at 48 h **a.-d.** Two-dimension scatter plots depicting distribution of cells positively stained for Annexin V-PE/7-AAD. (a) CG, (b) LG, (c) MG and (d) HG. Cells in lower left quadrant of each picture correspond to normal cells. Cells in right lower quadrant correspond to early apoptotic cells. Cells in right upper quadrant correspond to late apoptotic. Cells in left upper quadrant correspond to dead cells. **e**. Quantitative analysis of total apoptotic lymphocytes. Data are presented with the means ± standard deviation, * *p* < 0.05, ** *p* < 0.01, compared with the control group. Data were analyzed by the variance (ANOVA) test of the SPSS 19.0 software.

The flow cytometry assay showed that apoptotic lymphocytes were significantly increased (*p* < 0.01) in the MG and HG when compared with those in the LG and CG after NaF treatment for 24 h. And there was no significantly difference between LG and CG. However, with NaF treatment for 48 h, apoptotic lymphocytes were dramatically increased (*p* < 0.01) among NaF-treated groups and control group, and the apoptosis peaked at MG. The results were shown in Figure 6 and 7.

### NaF caused morphologic changes of apoptosis in splenic lymphocytes

**Figure 8:**
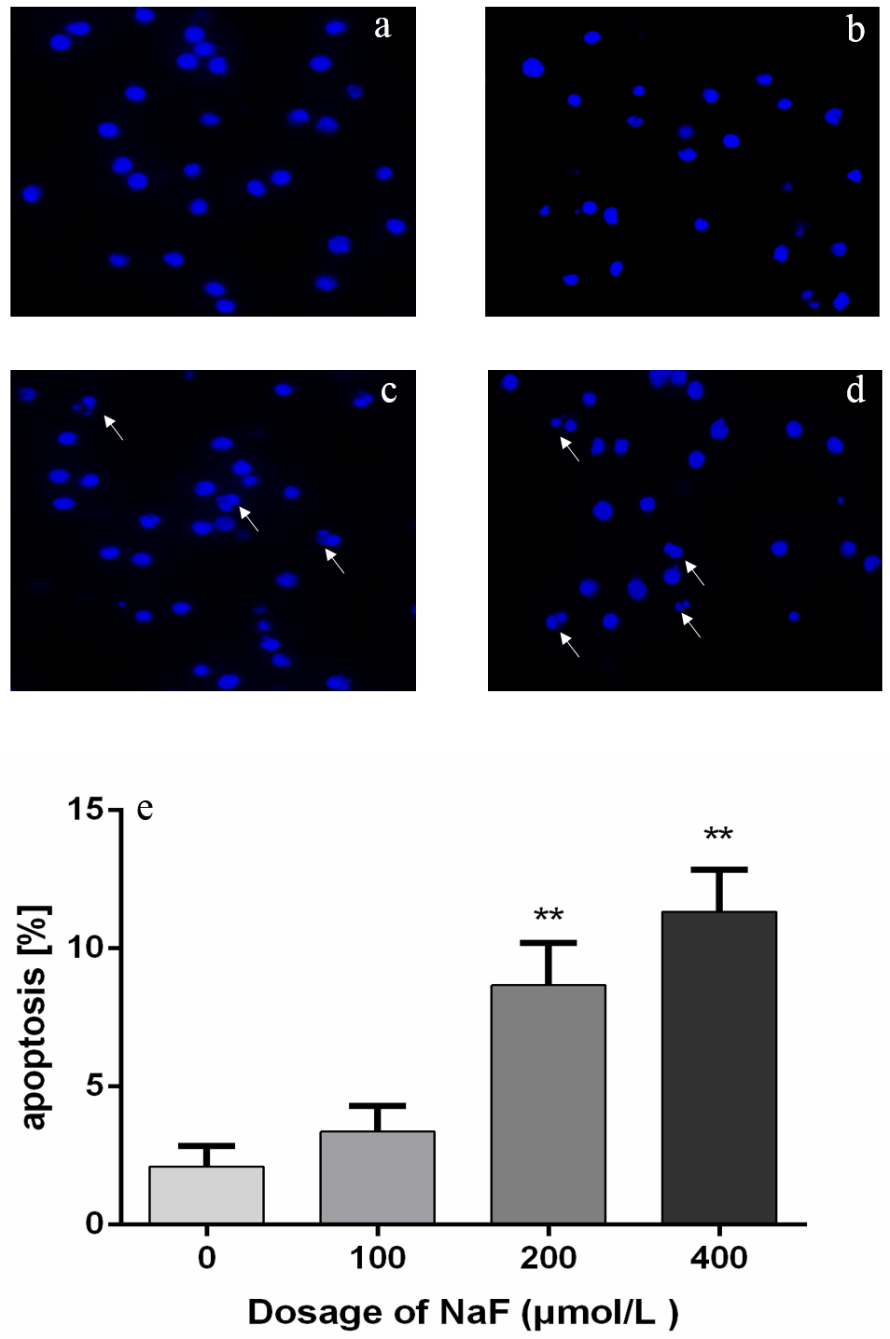
Effect of NaF treatment on morphologic changes of apoptosis in cultured splenic lymphocytes at 24 h **a.-d.** the nuclear morphology of lymphocytes stained with Hoechst 33258. (a) CG, (b) LG, (c) MG and (d) HG. Cells are observed under fuorescence microscope. × 1000. **e.** Quantitative analysis of apoptotic lymphocytes. Data are presented with the means ± standard deviation, * *p* < 0.05, ** *p* < 0.01, compared with the control group. Data were analyzed by the variance (ANOVA) test of the SPSS 19.0 software.

**Figure 9:**
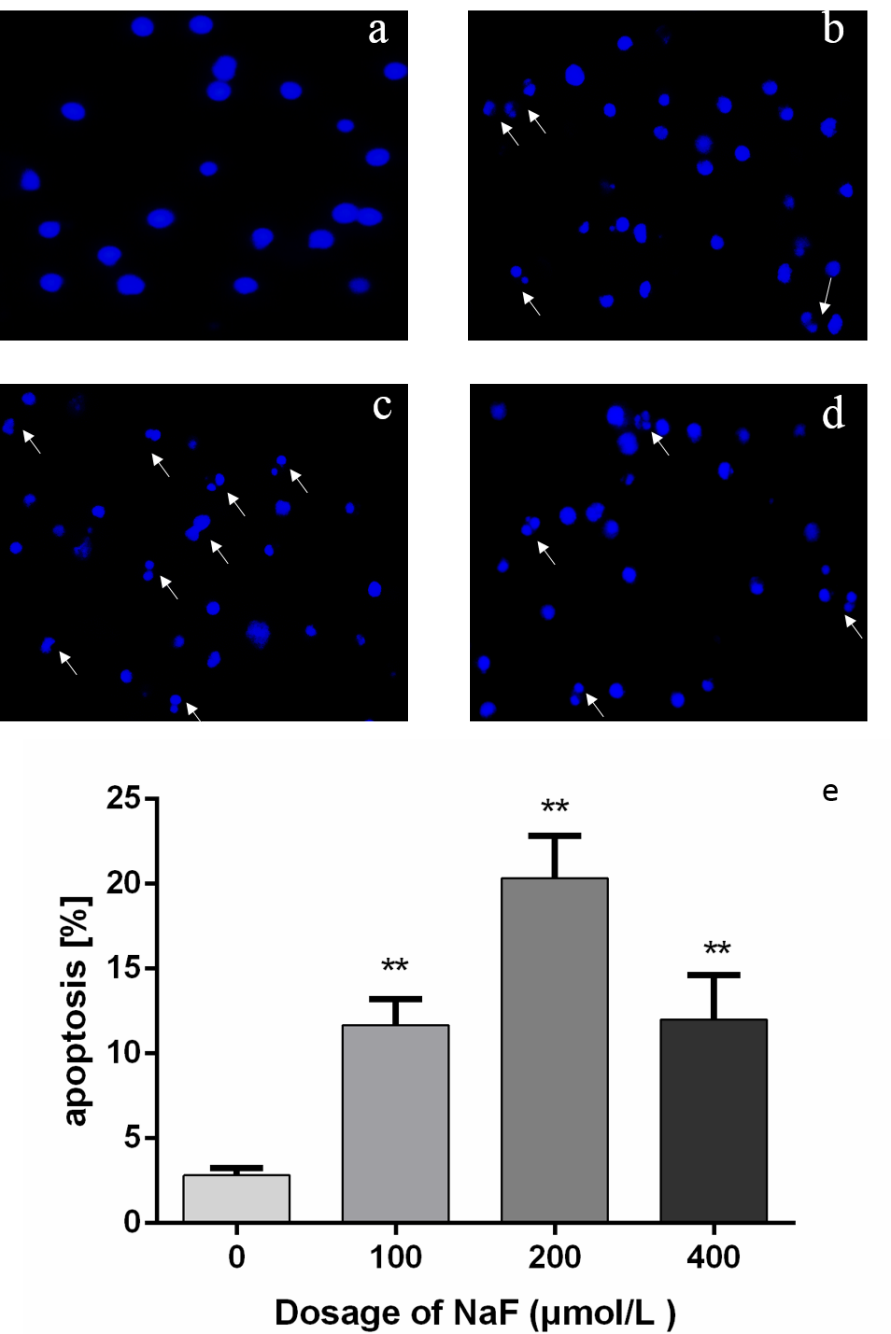
Effect of NaF treatment on morphologic changes of apoptosis in cultured splenic lymphocytes at 48 h **a.-d.** the nuclear morphology of lymphocytes stained with Hoechst 33258. (a) CG, (b) LG, (c) MG and (d) HG. Cells are observed under fuorescence microscope. × 1000. **e.** Quantitative analysis of apoptotic lymphocytes. Data are presented with the means ± standard deviation, * *p* < 0.05, ** *p* < 0.01, compared with the control group. Data were analyzed by the variance (ANOVA) test of the SPSS 19.0 software.

As shown in Figure 8 and 9, cells treated with NaF for 24 h and 48 h showed the characteristic features of apoptosis under a light microscope, including nuclear shrinkage and chromatin fragmenta­tion. Furthermore, the survival of the cells was significantly reduced. The number of apoptotic lymphocytes were significantly higher (*p* < 0.01) in the MG and HG than the LG and CG treated with NaF for 24 h. Also, exposed to NaF for 48 h, apoptotic lymphocytes were dramatically increased (*p* < 0.01) among NaF-treated groups and control group. Therefore, the short time treatment of NaF caused the typical apoptosis featured morphologic changes of splenic lymphocytes.

### Effects of NaF on caspase-9, -8, -7, -6 and -3 protein expression in splenic lymphocytes

**Figure 10:**
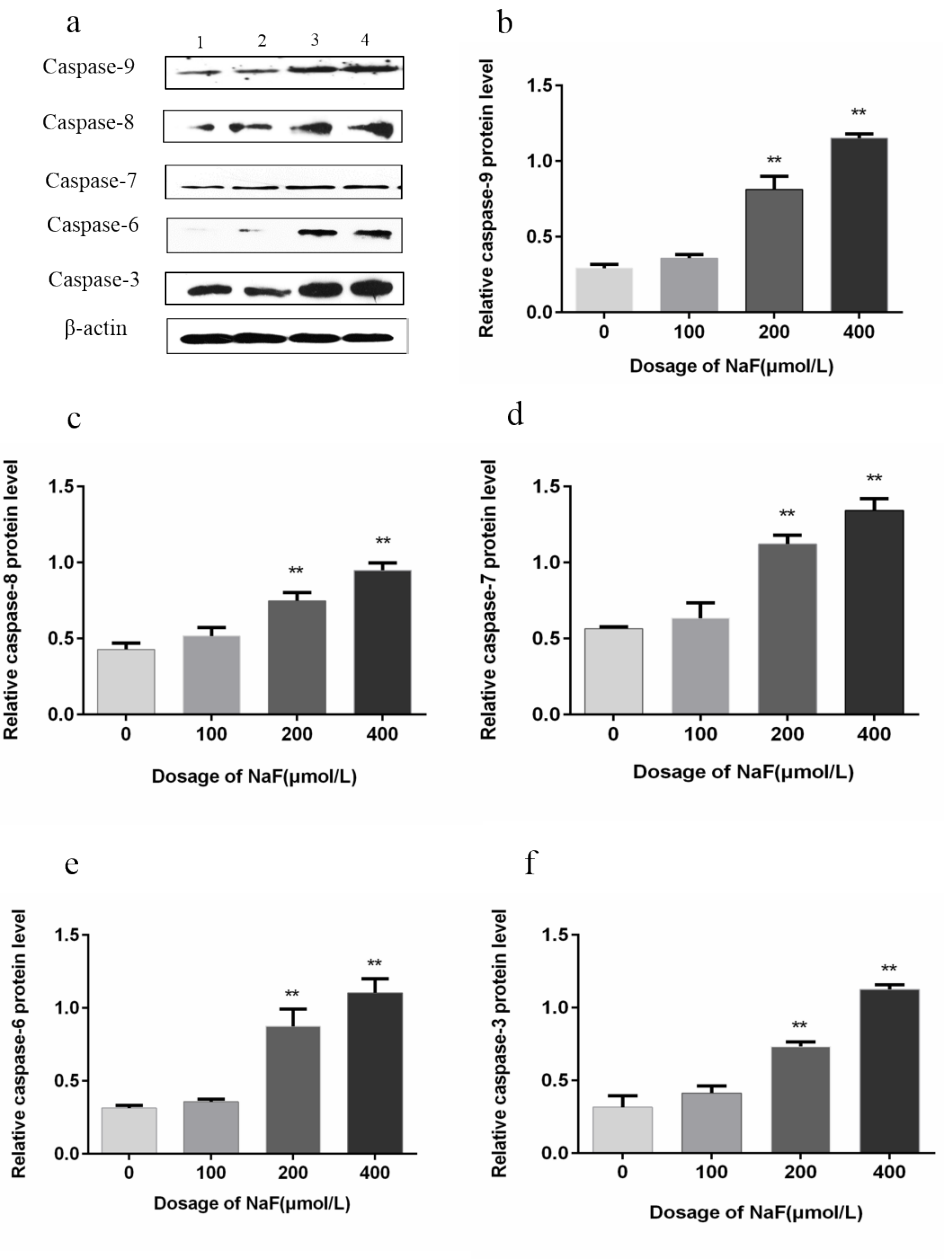
Effect of NaF treatment on protein expression levels of caspase-9, -8, -7, -6 and -3 in cultured splenic lymphocytes at 24 h **a.** The western blot assay. (1) CG, (2) LG, (3) LG and (4) HG. **b.-f.** Quantitative analysis of caspase-related proteins expression. Data are presented with the means ± standard deviation, * *p* < 0.05, ** *p* < 0.01, compared with the control group. Data were analyzed by the variance (ANOVA) test of the SPSS 19.0 software.

**Figure 11:**
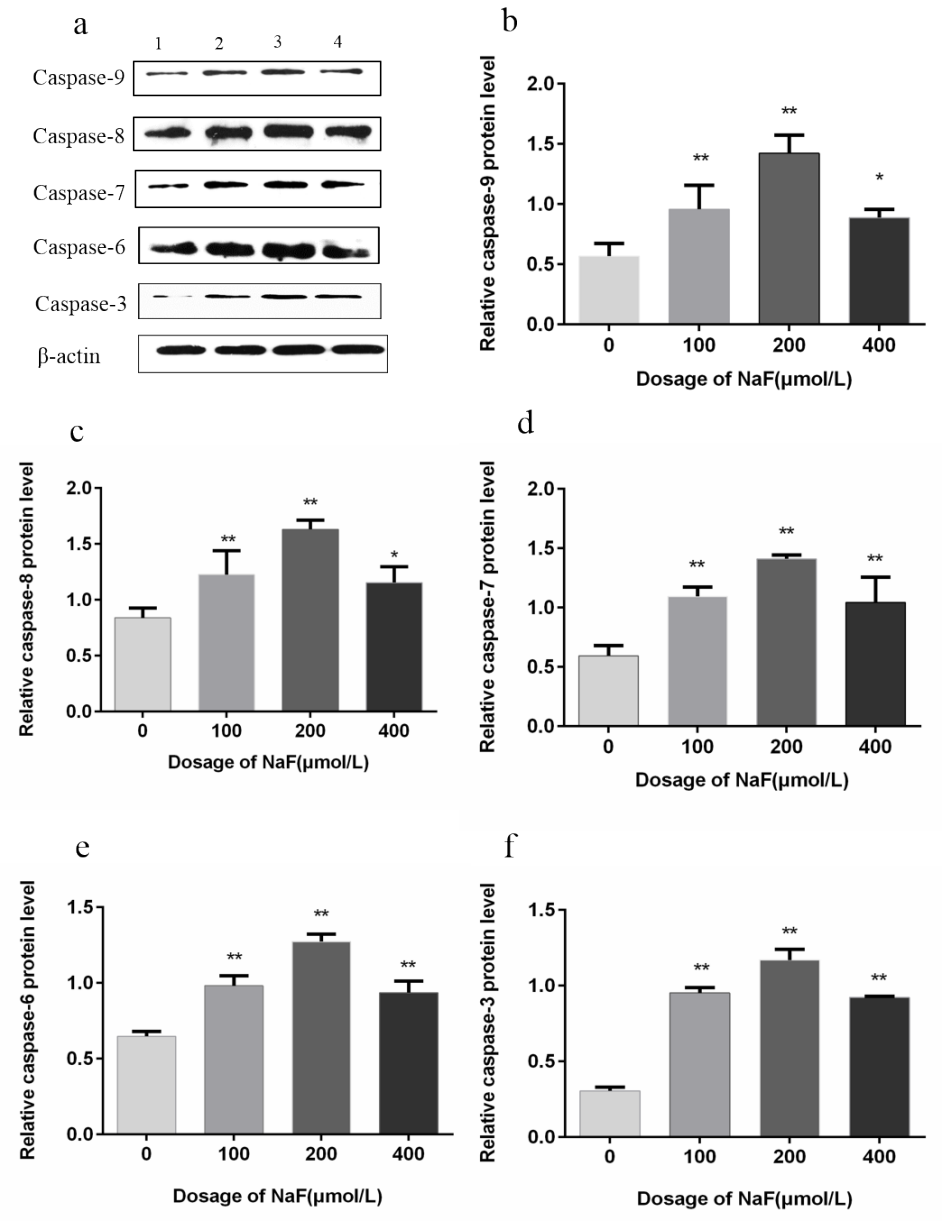
Effect of NaF treatment on protein expression levels of caspase-9, -8, -7, -6 and -3 in cultured splenic lymphocytes at 48 h **a.** The western blot assay. (1) CG, (2) LG, (3) MG and (4) HG. **b.**-**f.** Quantitative analysis of caspase-related proteins expression. Data are presented with the means ± standard deviation, * *p* < 0.05, ** *p* < 0.01, compared with the control group. Data were analyzed by the variance (ANOVA) test of the SPSS 19.0 software.

To further investigate the potential mechanisms involved in splenic lymphocytes apoptosis induced by NaF, protein expression levels of caspase-9, -8, -7, -6, -3 and -3 were measured by western blot. After NaF treatment for 24 h, the caspase-9, -8, -7, -6 and -3 protein expression levels were significantly increased (*p* < 0.01) in the MG and HG than those in the CG. After NaF treatment for 48 h, caspase-9, -8, -7, -6, -3 protein expression levels (*p* < 0.01 or *p* < 0.05) were significantly increased in the LG, MG and HG, and peaked in the MG when compared with those in the CG, which was consistent with the results of flow cytometry and Hoechst 33258 staining assay. The results were shown in Figure 10 and 11. Collectively, those data suggested that NaF-induced apoptosis could mediated by up-regulation of the above caspase family proteins.

### The effects of NaF on Bcl-2, Bcl-xL, Bax and Bak protein expression in splenic lymphocytes

**Figure 12:**
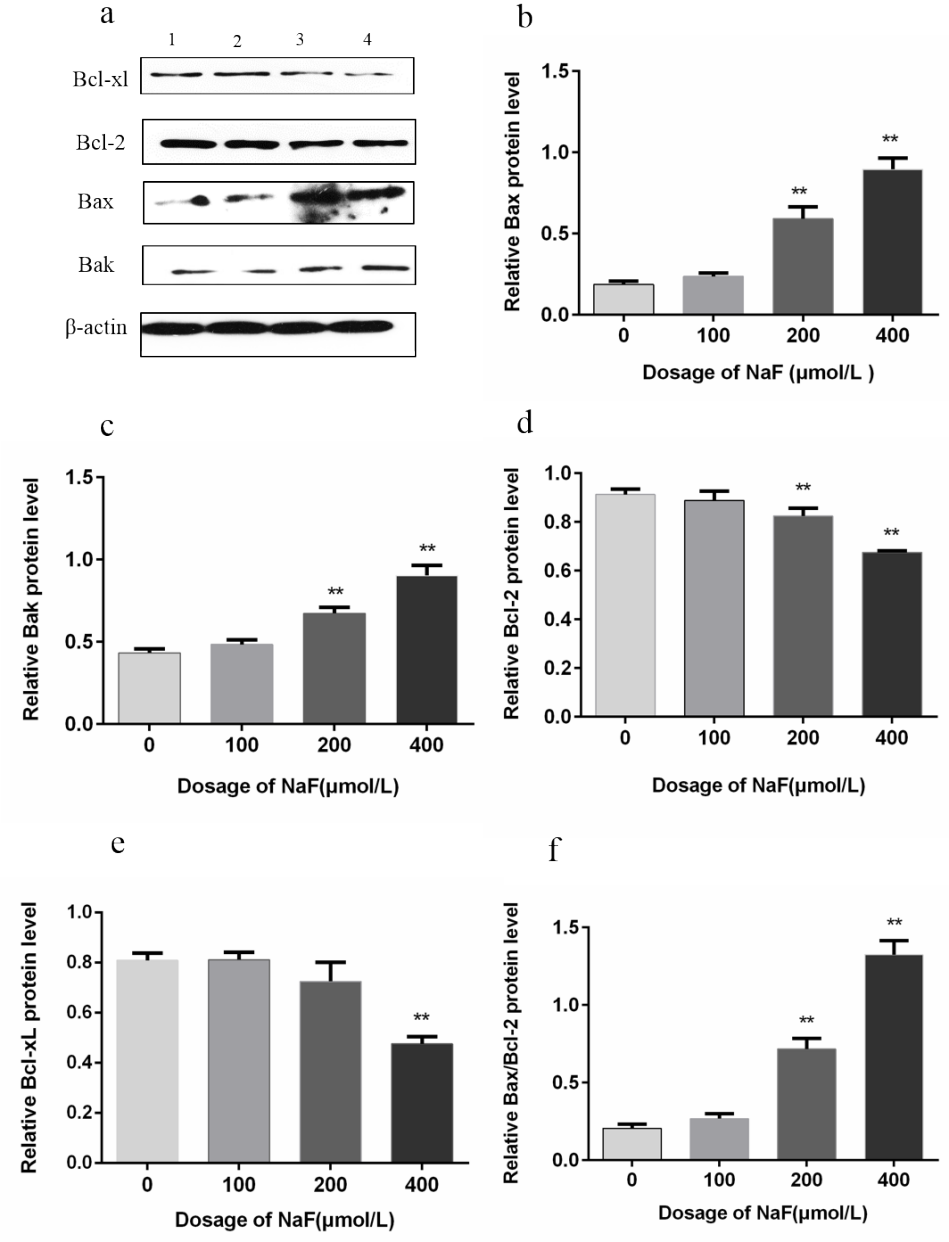
Effect of NaF treatment on protein expression levels of Bcl-2, Bcl-xL, Bax and Bak in cultured splenic lymphocytes at 24h **a.** The western blot assay. (1) CG, (2) LG, (3) MG and (4) HG. **b.**-**f.** Quantitative analysis of caspase-related proteins expression. Data are presented with the means ± standard deviation, * *p* < 0.05, ** *p* < 0.01, compared with the control group. Data were analyzed by the variance (ANOVA) test of the SPSS 19.0 software.

**Figure 13:**
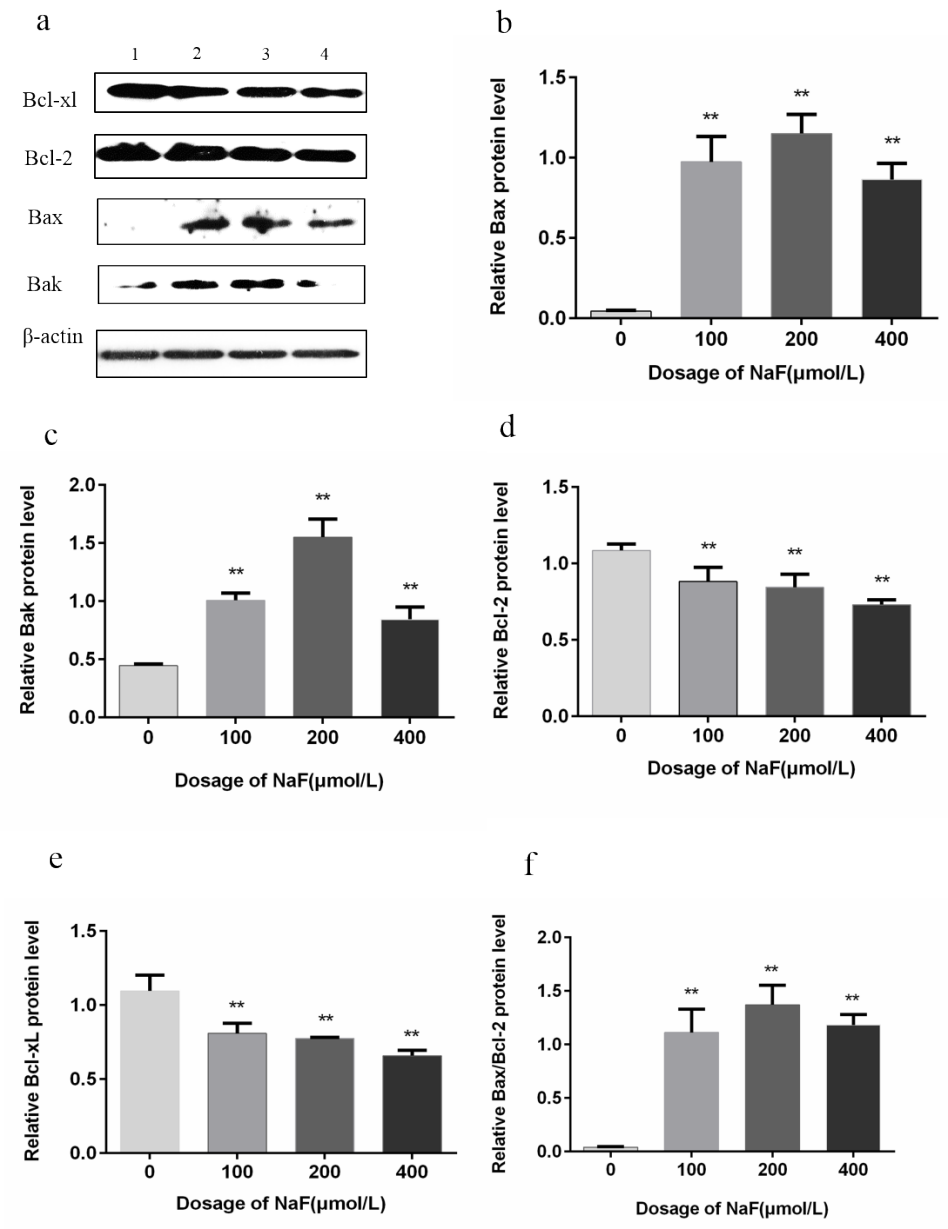
Effect of NaF treatment on protein expression levels of Bcl-2, Bcl-xL, Bax and Bak in cultured splenic lymphocytes at 48 h **a.** The western blot assay. (1) CG, (2) LG, (3) MG and (4) HG. **b.**-**f.** Quantitative analysis of caspase-related proteins expression. Data are presented with the means ± standard deviation, * *p* < 0.05, ** *p* < 0.01, compared with the control group. Data were analyzed by the variance (ANOVA) test of the SPSS 19.0 software.

The Bcl-2 family members play an important role in cell apoptosis and survival. As shown in Figure 12, Bax and Bak protein expression levels were significantly increased (*p* < 0.01) in the MG and HG in comparison with control group after NaF treatment for 24 h. Bcl-2 protein expression levels were significantly reduced (*p* < 0.01) in the MG and HG, and Bcl-xL protein levels were decreased (*p* < 0.01) in the HG. Also, Bax/Bcl-2 ratio was markedly increased (*p* < 0.01) in the MG and HG after NaF treatment for 24 h. Meanwhile, protein expression levels of Bax and Bak were higher (*p* < 0.01) in the LG, MG and HG than those in the CG after NaF treatment for 48 h. Bcl-2 and Bcl-xL protein expression levels were decreased (*p* < 0.01) in the LG, MG and HG (Figure 13). The Bax/Bcl-2 ratio was also increased (*p* < 0.01) in the NaF-treated groups after NaF treatment for 48 h.

### Effects of NaF on Fas and FasL protein expression in splenic lymphocytes

**Figure 14:**
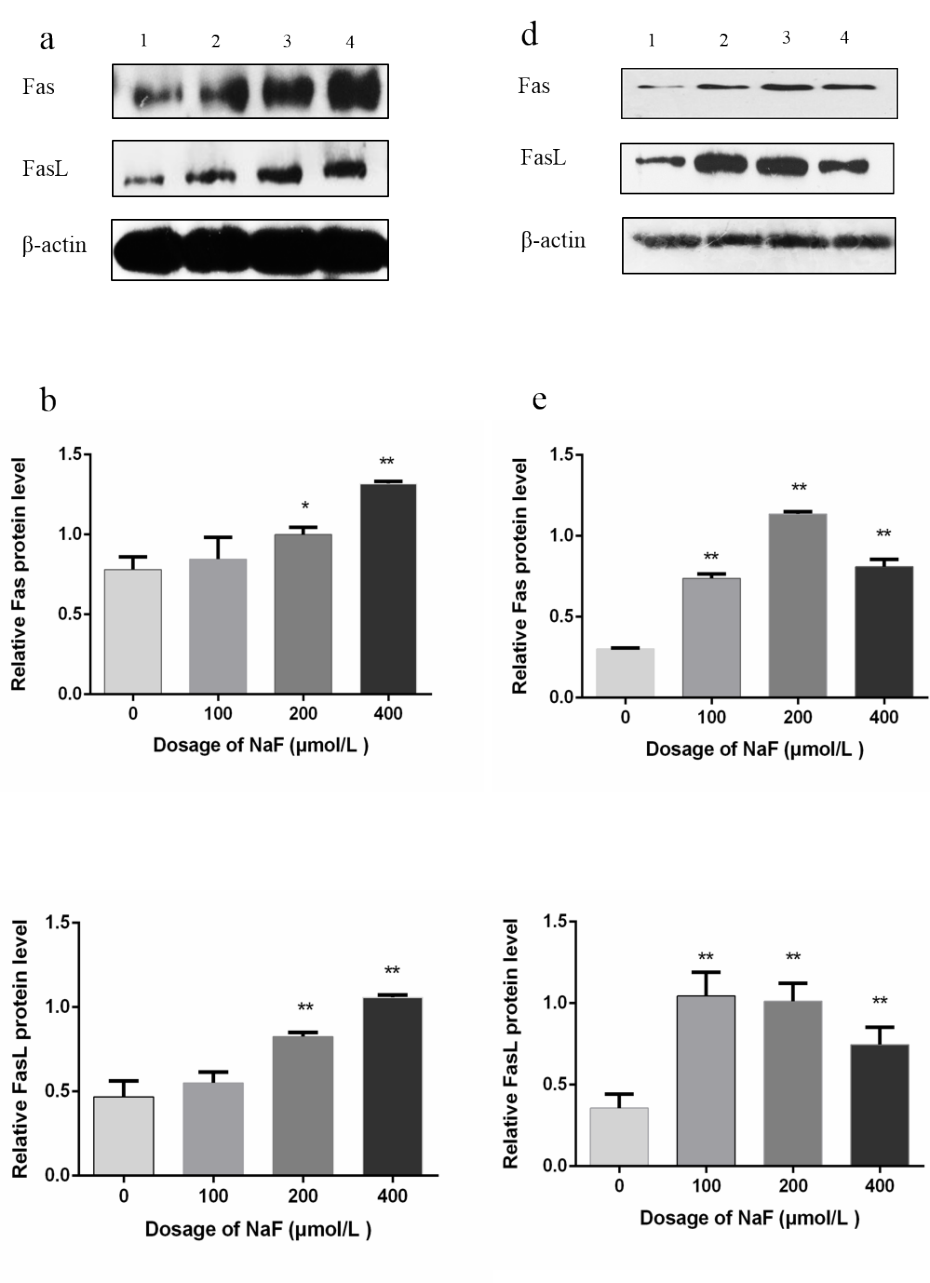
Effect of NaF treatment on protein expression levels of Fas and FasL in cultured splenic lymphocytes at 24 (a-c) and 48 h (d-f) **a.**, **d.** The western blot assay. (1) CG, (2) LG, (3) MG and (4) HG. **b.**, **c.**, **e.**, **f.** Quantitative analysis of caspase-related proteins expression. Data are presented with the means ± standard deviation, * *p* < 0.05, ** *p* < 0.01, compared with the control group. Data were analyzed by the variance (ANOVA) test of the SPSS 19.0 software.

Fas and FasL are important molecules in the extrinsic apoptosis pathway, As shown in Figure 14a-14c, the protein expression levels of Fas and FasL were significantly higher (*p* < 0.01 or *p* < 0.05) in the MG and HG than those in the CG after NaF treatment for 24 h. After NaF treatment for 48 h, the Fas and FasL protein expression levels were significantly increased (*p* < 0.01) in the LG, MG and HG, and peaked in the MG when compared with those in the CG (Figure 14d-14f). Thus, the NaF caused the cell apoptosis through death receptor Fas/FasL pathway.

## DISCUSSION

It is well known that fluoride have diverse effects on cells. In bone cells, fluorides elicited potentially beneficial effects by stimulating bone cell growth [6]. However, in other cultured cells, NaF can alter cellular metabolism [44], induce inflammatory cytokines production [40], inhibit protein secretion and synthesis [45], and influence signaling pathways involved in proliferation and apoptosis [46-48]. In this *in vitro* study, flow cytometry assay and Hoechst 33258 staining showed that NaF induced apoptosis in splenic lymphocytes from mice. It is well known that up-regulation of pro-apoptotic or down-regulation of anti-apoptotic proteins can lead to cell death. To reveal the pathway involved in NaF-induced apoptosis, we detected the ROS production, mitochondria transmembrane potential (MMP), protein expression of caspases, Fas, FasL and Bcl-2 family members.

Previous report has demonstrated that oxidative stress is induced in fluoride group in human blood lymphocytes and lead apoptosis [49]. However, our results showed that NaF did not change intracellular concentration of ROS, indicating that NaF-induced apoptosis of mouse splenic lymphocytes was independent of ROS production.

The mitochondrial pathway, which is controlled by Bcl-2 family members, is the most important intracellular apoptosis signaling cascade. Bcl-2 family, as a group of crucial regulatory factors in apoptosis, can indirectly regulate the activity of caspases in related apoptotic pathways [50]. Members of Bcl-2 family are classified into two groups: pro-apoptotic proteins and anti-apoptotic proteins. Pro-apoptotic proteins such as Bax and Bak induce apoptosis by causing loss of the mitochondrial membrane potential [51, 52]. Anti-apoptotic proteins such as Bcl-2 and Bcl-xL can maintain the normal permeability of mitochondrial permeability transition pore (MPTPs) and block the release of mitochondrial proapoptotic factor by binding selectively to the active conformation of Bax. Thus, the anti-apoptosis protein prevents cell apoptosis [52-54]. Yang et al. has reported that fluoride induce apoptosis in matured ameloblast-like LS8 cells by down-regulating Bcl-2 [55]. The similar picture has been observed in the NaF-treated renal tubules [56] and osteoblastic MC3T3-E1 cells [57], where the apoptosis induced by NaF is also associated with the down-regulation of Bcl-2 and up-regulation of Bax expression at both the mRNA and protein levels. In the present study, Bcl-2 and Bcl-xL was shown to be down-regulated whereas Bax and Bak were up-regulated in lymphocytes treated with NaF and an increase in the Bax/Bcl-2 ratio was also observed.

The mitochondrial-mediated apoptotic pathway is accompanied by Δ*Ψm* depolarization, followed by cytochrome *c* release from mitochondria into the cytosol. After cytochrome c is released from mitochondria to the cytosol, caspase-9 is subsequently activated through binding to the CED-4 homolog Apaf-1 [58], and then caspase-3, -6 and -7 are activated [59]. Once executioner caspases such as caspase-3, -6 and -7 are activated, they subsequently cleave distinct cellular proteins to cause apoptosis [42, 60]. In the present paper, a decrease in Δ*Ψm* was detected in the NaF-treated cells, together with the increased protein expression of caspase-3, -6, -7 and -9. Based on the above-mentioned data, mitochondria dependent apoptotic pathway may be involved in NaF-induced apoptosis in lymphocytes.

Most recent researches regarding apoptotic signaling cascades support a model in which two caspase generated pathways ensure cell death [61]. In the extrinsic apoptosis pathway, interaction of Fas and FasL lead to the activation of Caspase-8 [62]. Activated Caspase-8 can directly cleave and activate downstream effector proteases, such as Caspase-3, -6, -7, causing apoptosis [63]. In order to assess whether there is any role of the extrinsic apoptosis pathway in the activation of caspase-3, which is a key factor in apoptosis execution of both extrinsic and intrinsic pathways, we measured the protein levels of Fas, FasL and caspase-8. We found that they were also significantly up-regulated after NaF treatment. Therefore, the present study demonstrates that death receptor-mediated pathway may be also involved in NaF-induced apoptosis of lymphocytes. These results further suggest that NaF-induced apoptosis may occur *via* both mitochondria- and death receptor-dependent apoptosis pathways.

The spleen is a peripheral immune organ and plays an important role in maintaining immune homeostasis [64-66]. It is mainly composed of lymphocytes, which are involved in cellular immunity and humoral immunity. This *in vitro* study has proved that NaF-induced apoptosis in splenic lymphocytes, which impacts lymphocyte numbers and activities. Thus, the splenic immune function has finally been impaired due to the increased lymphocyte apoptosis and decreased lymphocyte numbers and activities.

## MATERIALS AND METHODS

### Chemicals and supplies

NaF (S6776) was purchased from Sigma Aldrich, UK. Lymphocyte separation medium (DKW33-R0100) were supplied by Dakewe Biotech Company, China. JC-1 mitochondrial membrane potential assay kit (C2006), ROS Assay kit (S0033), RIPA lysis buffer (P0013C), BCA Protein Assay Kit (P0012), Cell Counting Kit-8 (CCK-8) (C0038) and Hoechst 33258 (C1011) were obtained from Beyotime Biotechnology, China. RPMI 1640 (11875119) and fetal bovine serum (16000044) were supplied by Gibco, UK. The mouse Bc1-2 (3498T), Bcl-xl (2764T), Bax (14796S), Bak (12105T), caspase-9 (9508T), cleaved caspase-8 (8592T), cleaved caspase-7 (8438S), cleaved caspase-6 (9761T), cleaved caspase-3 (9664T) antibodies, rabbit IgG (7074P2) and mouse IgG (7076P2) were obtained from Cell Signaling Technology, UK. Fas (ab82419) and FasL (ab16104) were purchased from Abcam, UK. All other reagents used were analytical grade.

### Lymphocyte isolation, culture and treatment

The ICR mice were obtained from the Experimental Animal Corporation of DOSSY Biological Technology Company. 3-week-old ICR male mice were anaesthetized and euthanized. After laparotomy, the spleen was separated from the mice and washed with cold phosphate buffered saline (PBS, pH 7.4). Then, the spleen was placed in a 200-mesh stain steel sieve over a culture dish containing 4-5 mL lymphocyte isolation separation medium and grounded into small pieces with the plunger of glass syringe. The liquid was transferred into a centrifuge tube, and then 200-500 μL RPMI-1640 medium was added and centrifuged at 800×*g* for 30 min at room temperature. Three layers were formed after centrifugation. The middle milky layer which contained lymphocytes was transferred into a test tube. The lymphocytes were washed twice with PBS and suspended in RPMI-1640 medium with 10 % fetal calf serum and then transferred into a culture bottle. All these processes were performed under sterile condition. The viability of the lymphocyte was estimated according to the trypan blue exclusion criteria, and the viability is over 95%.

To monitor various parameters (except the CCK-8 bioas-say), splenic lymphocytes were cultured in the RPMI-1640 medium (supplemented with 10% fetal calf serum, 100 U/mL penicillin, 100 μg/mL streptomycin) containing 0 (control group, CG), 100(low-dose group, LG), 200 (medial-dose group, MG), and 400 (high-dose group, HG) μmol/L NaF. Triplicates were performed in each treatment. All cells were maintained in a humidified incubator for 24 and 48 h at 37 °C with 5% CO2.

Our experiments involving the use of mice and all experimental procedures were approved by Animal Care and Use Committee, Sichuan Agricultural University.

### Measurement of cell viability

Cell viability was assessed using the CCK-8 bioassay [67, 68]. Splenic lymphocytes (7×10^5^/well) were seeded into 96-well flat-bottomed plates and were exposed to NaF (0-1400 μmol/L) for 12 h, 24 h, 36 h, 48 h and 72 h at 37 °C with 5% CO2. After NaF exposure, 10 μL CCK-8 solutions were added to each well, and incubated for 4 h. The optical density (OD) was measured at 450 nm using a microplate reader.

### Detection of ROS production

The ROS production was evaluated by using an ROS detection Kit according to the manufacturer's protocol. Cells were collected by centrifugation and washed in PBS at 800 g for 5 min, and then resuspended in 100 μl PBS. Thereafter, cells stained with 10 μM DCFH-DA for 20 min at 37°C. Then, 400 μL PBS was added. The Rosup (a compound mixture) was used as a positive control. Data were then obtained by a FACSCalibur (Becton Dickinson, USA).

### Measurement of mitochondria transmembrane potential (MMP)

JC-1 was used to determine mitochondrial membrane potential (Δψm). Cells were collected and transferred into 5 mL culture tube, and then centrifuged. Afterwards, 0.5 mL 1×JC-1 working solution was added and then incubated in the mixture for 20 min at 37 °C under 5% CO_2_ incubator. At the end of the incubations, cells were washed twice with 1× JC-1 Assay Buffer, and then resuspended each pellet in 450 μL 1× JC-1Assay Buffer. Finally, Δψm was assayed by FCM within 30 minutes [69].

### Measurement of apoptosis

The percentage of apoptosis was evaluated by an Annexin V-PE/7-AAD staining detection Kit (BD Biosciences, San Jose, CA, USA) according to the manufacturer's protocol. Cells were cultured in the presence of 0, 100, 200 and 400 μmol/L NaF for 24 h and 48 h. Prior to toxicity detection, the cells were collected and washed two times with phosphate buffered saline (PBS, PH 7.4). Pellets were collected and resuspended in 100 μL PBS, and stained with PE Annexin V and 7-amino-actinomycin (7-AAD) for 15 minutes in the dark. Then, 400 μL binding buffer (BD Pharmingen) was added. Data were then obtained by a FACSCalibur (Becton Dickinson, USA).

### Hoechst 33258 staining

Cells were collected in 1.5 ml centrifuge tubes, washed with PBS and fixed with 4% paraformaldehyde for 10 min. And then centrifuged at 2000×*g* for 5 min. Cells were then washed with PBS for two times again and stained with Hoechst 33258 (Beyotime, Jiangsu, China) at 10 μg/mL for 10 minutes in dark. After washed with PBS, the cells were added to the glass slide and then covered with a clean cover glass. Nuclear morphology was observed using fluorescence microscopy. Apoptotic cells were identified by the reduction in the volume, chromatin condensation and fragmentation of their nuclei [70-72]. The percentage of apoptotic cells was calculated from ten randomly selected microscopic fields (1000×magnification).

### Western blot analysis

Cells were lysed and proteins were extracted with RIPA lysis buffer and then kept in laemmli buffer. Protein samples were separated by SDS-PAGE (10%-15% gels) and transferred to nitrocellulose filter membranes. The membranes were blocked in 5% nonfat dry milk for 1 h and then incubated with the primary antibodies overnight at 4°C. The primary antibodies were Bax, Bak, Bcl-2, Bcl-xL, Fas, FasL and caspase-3, -6, -7, -8, -9. The membranes were then washed with PBS-Tween (PBST) and incubated with the biotin-conjugated secondary antibodies for 1 h, and washed again with PBS-Tween (PBST). Blots were visualized by ECL™ (Bio-Rad) and X-ray film. When the band is thicker and darker, protein expression is higher, otherwise it is lower. Then, the statistical data of protein expression was done with ImageJ2x software

### Statistical analysis

All the data were analyzed by SPSS 19.0. All the results were expressed as mean±SD. Data were analyzed by one way analysis of variance (ANOVA). *p* < 0.05 was considered as a significant difference.

### Conclusions

In summary, 200 μmol/L and 400 μmol/L NaF induce the cultured splenic lymphocytes apoptosis, which is promoted by up-regulating of Bax, Bak, caspase-9, caspase-8, caspase-7, caspase-6 and caspase-3 protein expression, and down-regulating of mitochondria membrane potential, Bcl-2 and Bcl-xL protein expression. Also, the effect of NaF on cultured splenic lymphocytes apoptosis shows time and dose dependent.
